# 1-(1,5-Diphenyl-4-phenyl­sulfonyl-1*H*-pyrazol-3-yl)ethanone

**DOI:** 10.1107/S1600536812035027

**Published:** 2012-08-15

**Authors:** Hoong-Kun Fun, Ching Kheng Quah, Hatem A. Abdel-Aziz, Hazem A. Ghabbour

**Affiliations:** a X-ray Crystallography Unit, School of Physics, Universiti Sains Malaysia, 11800 USM, Penang, Malaysia; b Department of Pharmaceutical Chemistry, College of Pharmacy, King Saud University, PO Box 2457, Riyadh 11451, Saudi Arabia

## Abstract

The asymmetric unit of the title compound, C_23_H_18_N_2_O_3_S, contains two mol­ecules with comparable geometries. In one mol­ecule, the pyrazole ring forms dihedral angles of 61.65 (11), 47.88 (11) and 63.20 (14)° with the three benzene rings. The corresponding values for the other mol­ecule are 77.19 (11), 43.55 (11) and 63.56 (15)°. In the crystal, both mol­ecules are linked into inversion dimers by pairs of C—H⋯S hydrogen bonds, generating *R*_2_^2^(14) loops in each case.

## Related literature

For background to and pharmaceutical applications of pyrazole derivatives, see: Gürsoy *et al.* (2000 ▶); Kurumbail *et al.* (1996 ▶). For further synthetic details, see: Saleh & Abd El-Rahman (2009 ▶); Nassar *et al.* (2011 ▶).
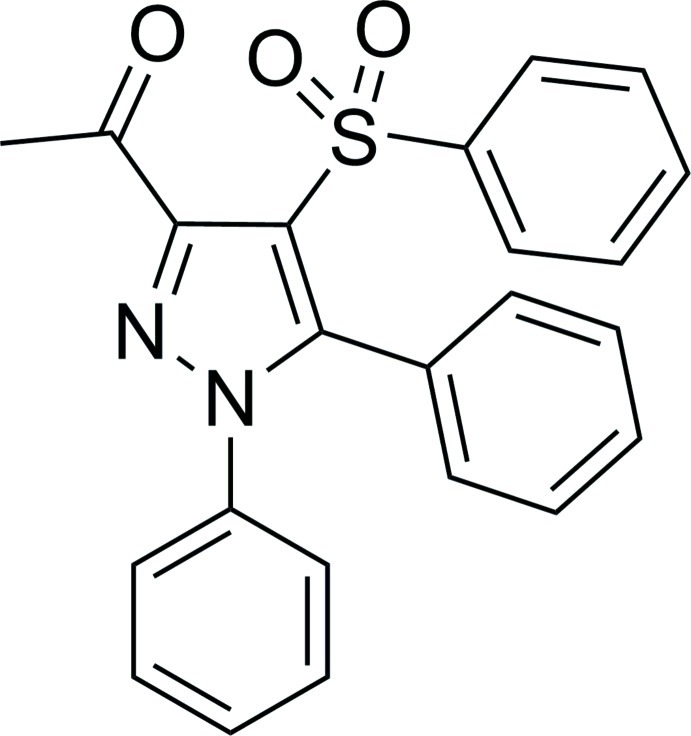


## Experimental

### 

#### Crystal data

C_23_H_18_N_2_O_3_S
*M* *_r_* = 402.45
Triclinic, 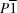

*a* = 10.4078 (2) Å
*b* = 14.0839 (3) Å
*c* = 14.2468 (3) Å
α = 87.595 (2)°
β = 80.875 (2)°
γ = 86.850 (2)°
*V* = 2057.62 (7) Å^3^
*Z* = 4
Cu *K*α radiation
μ = 1.62 mm^−1^
*T* = 296 K
0.66 × 0.55 × 0.17 mm

#### Data collection

Bruker SMART APEXII CCD diffractometer
Absorption correction: multi-scan (*SADABS*; Bruker, 2009 ▶) *T*_min_ = 0.415, *T*_max_ = 0.771
19520 measured reflections
6561 independent reflections
5618 reflections with *I* > 2σ(*I*)
*R*_int_ = 0.029

#### Refinement

*R*[*F* ^2^ > 2σ(*F* ^2^)] = 0.042
*wR*(*F* ^2^) = 0.123
*S* = 1.05
6561 reflections
525 parameters
H-atom parameters constrained
Δρ_max_ = 0.30 e Å^−3^
Δρ_min_ = −0.31 e Å^−3^

### 

Data collection: *APEX2* (Bruker, 2009 ▶); cell refinement: *SAINT* (Bruker, 2009 ▶); data reduction: *SAINT*; program(s) used to solve structure: *SHELXTL* (Sheldrick, 2008 ▶); program(s) used to refine structure: *SHELXTL*; molecular graphics: *SHELXTL*; software used to prepare material for publication: *SHELXTL* and *PLATON* (Spek, 2009 ▶).

## Supplementary Material

Crystal structure: contains datablock(s) global, I. DOI: 10.1107/S1600536812035027/hb6911sup1.cif

Structure factors: contains datablock(s) I. DOI: 10.1107/S1600536812035027/hb6911Isup2.hkl

Supplementary material file. DOI: 10.1107/S1600536812035027/hb6911Isup3.cml

Additional supplementary materials:  crystallographic information; 3D view; checkCIF report

## Figures and Tables

**Table 1:** Hydrogen-bond geometry (Å, °)

*D*—H⋯*A*	*D*—H	H⋯*A*	*D*⋯*A*	*D*—H⋯*A*
C11*A*—H11*A*⋯O2*A* ^i^	0.93	2.59	3.428 (3)	149
C11*B*—H11*B*⋯O3*B* ^ii^	0.93	2.57	3.370 (3)	144

Symmetry codes: (i) 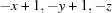; (ii) 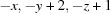.

**Fig. 1.**
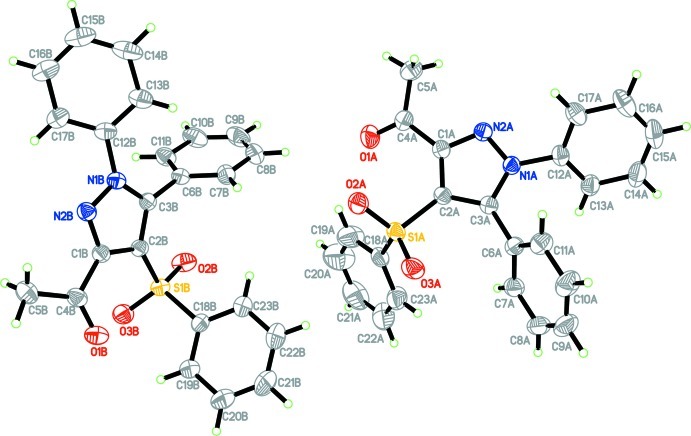
The asymmetric unit of the title compound showing 30% probability displacement ellipsoids for non-H atoms.

**Fig. 2.**
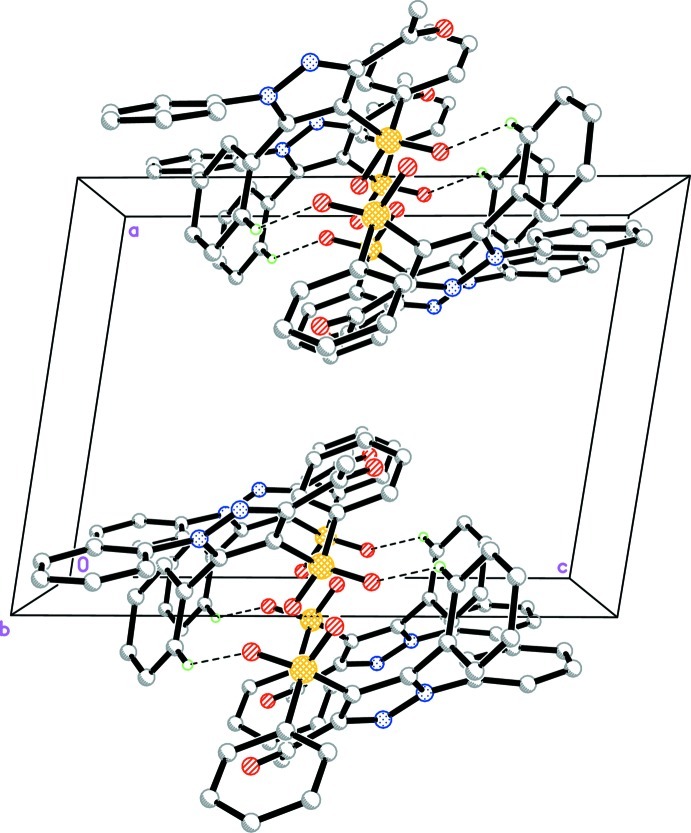
The crystal structure of the title compound, viewed along the b axis. H atoms not involved in hydrogen bonds (dashed lines) have been omitted for clarity.

## supplementary crystallographic information

### Comment

Pyrazole derivatives have been attracted a great deal of research interest because of their various pharmaceutical applications (Gürsoy *et al.*, 2000). *N*-arylpyrazoles were found to be a main pharmacophore in the famous anti-inflammatory drugs Celecoxib and SC-558 (Kurumbail *et al.*, 1996). As part of our atudies in this area, we now describe the crystal structure of the title compound.

The asymmetric unit (Fig. 1) of the title compound consists of two independent molecules (*A* and *B*), with comparable geometries. In molecule *A*, pyrazol-3-yl ring (N1A/N2A/C1A-C3A) forms dihedral angles of 61.65 (11), 47.88 (11) and 63.20 (14)° with the three benzene rings (C6A-C11A, C12A-C17A, C18A-C23A), respectively. The corresponding dihedral angles for molecule *B* are 77.19 (11), 43.55 (11) and 63.56 (15)°, respectively.

In the crystal, Fig. 2, both independent molecules are linked into inversion dimers by pairs of C—H···S hydrogen bonds (Table 1), generating *R*_2_^2^(14) loops in each case.

### Experimental

The title compound was prepared by the reaction of (*Z*)-2-oxo-*N'*-phenylpropanehydrazonoyl chloride with 1-phenyl-2-(phenylsulfonyl)ethanone according to the reported method (Saleh & Abd El-Rahman, 2009; Nassar *et al.*, 2011). Yellow plates were obtained by slowly evaporating an ethanol solution at room temperature.

### Refinement

All H atoms were positioned geometrically and refined using a riding model with C–H = 0.93 or 0.96 Å and *U*_iso_(H) = 1.2 or 1.5 *U*_eq_(C). A rotating-group model was applied for the methyl groups.

### Crystal data

**Table:**

C_23_H_18_N_2_O_3_S	*Z* = 4
*M**_r_* = 402.45	*F*(000) = 840
Triclinic, *P*1	*D*_x_ = 1.299 Mg m^−^^3^
Hall symbol: -P 1	Cu *K*α radiation, λ = 1.54178 Å
*a* = 10.4078 (2) Å	Cell parameters from 2375 reflections
*b* = 14.0839 (3) Å	θ = 3.1–69.6°
*c* = 14.2468 (3) Å	µ = 1.62 mm^−^^1^
α = 87.595 (2)°	*T* = 296 K
β = 80.875 (2)°	Plate, yellow
γ = 86.850 (2)°	0.66 × 0.55 × 0.17 mm
*V* = 2057.62 (7) Å^3^	

### Data collection

**Table:**

Bruker SMART APEXII CCD diffractometer	6561 independent reflections
Radiation source: fine-focus sealed tube	5618 reflections with *I* > 2σ(*I*)
Graphite monochromator	*R*_int_ = 0.029
φ and ω scans	θ_max_ = 64.0°, θ_min_ = 3.1°
Absorption correction: multi-scan (*SADABS*; Bruker, 2009)	*h* = −12→12
*T*_min_ = 0.415, *T*_max_ = 0.771	*k* = −16→15
19520 measured reflections	*l* = −16→16

### Refinement

**Table:**

Refinement on *F*^2^	Primary atom site location: structure-invariant direct methods
Least-squares matrix: full	Secondary atom site location: difference Fourier map
*R*[*F*^2^ > 2σ(*F*^2^)] = 0.042	Hydrogen site location: inferred from neighbouring sites
*wR*(*F*^2^) = 0.123	H-atom parameters constrained
*S* = 1.05	*w* = 1/[σ^2^(*F*_o_^2^) + (0.079*P*)^2^ + 0.2166*P*] where *P* = (*F*_o_^2^ + 2*F*_c_^2^)/3
6561 reflections	(Δ/σ)_max_ = 0.001
525 parameters	Δρ_max_ = 0.30 e Å^−^^3^
0 restraints	Δρ_min_ = −0.31 e Å^−^^3^

### Special details

**Table:**

Geometry. All esds (except the esd in the dihedral angle between two l.s. planes) are estimated using the full covariance matrix. The cell esds are taken into account individually in the estimation of esds in distances, angles and torsion angles; correlations between esds in cell parameters are only used when they are defined by crystal symmetry. An approximate (isotropic) treatment of cell esds is used for estimating esds involving l.s. planes.
Refinement. Refinement of F^2^ against ALL reflections. The weighted R-factor wR and goodness of fit S are based on F^2^, conventional R-factors R are based on F, with F set to zero for negative F^2^. The threshold expression of F^2^ > 2sigma(F^2^) is used only for calculating R-factors(gt) etc. and is not relevant to the choice of reflections for refinement. R-factors based on F^2^ are statistically about twice as large as those based on F, and R- factors based on ALL data will be even larger.

### Fractional atomic coordinates and isotropic or equivalent isotropic displacement parameters (Å^2^)

**Table:**

	*x*	*y*	*z*	*U*_iso_*/*U*_eq_	
S1A	0.37925 (5)	0.47575 (3)	0.16765 (3)	0.05013 (14)	
O1A	0.13209 (17)	0.55466 (13)	0.04899 (13)	0.0789 (5)	
O2A	0.36657 (18)	0.57364 (10)	0.13967 (11)	0.0690 (4)	
O3A	0.49637 (15)	0.44031 (12)	0.20008 (11)	0.0684 (4)	
N1A	0.39837 (14)	0.30565 (11)	−0.04242 (11)	0.0463 (3)	
N2A	0.31322 (14)	0.37098 (11)	−0.07247 (11)	0.0490 (4)	
C1A	0.28797 (17)	0.43418 (13)	−0.00471 (13)	0.0469 (4)	
C2A	0.35983 (17)	0.40841 (13)	0.07040 (13)	0.0451 (4)	
C3A	0.43075 (17)	0.32531 (13)	0.04339 (13)	0.0452 (4)	
C4A	0.19157 (19)	0.51359 (14)	−0.01795 (16)	0.0553 (5)	
C5A	0.1720 (2)	0.5387 (2)	−0.11806 (19)	0.0762 (7)	
H5AA	0.1036	0.5874	−0.1177	0.114*	
H5AB	0.1486	0.4833	−0.1472	0.114*	
H5AC	0.2513	0.5616	−0.1534	0.114*	
C6A	0.52929 (18)	0.26530 (13)	0.08549 (14)	0.0501 (4)	
C7A	0.4967 (2)	0.21668 (15)	0.17240 (15)	0.0617 (5)	
H7AA	0.4122	0.2222	0.2053	0.074*	
C8A	0.5903 (3)	0.16043 (17)	0.20940 (19)	0.0799 (8)	
H8AA	0.5688	0.1285	0.2678	0.096*	
C9A	0.7151 (3)	0.1510 (2)	0.1608 (2)	0.0911 (9)	
H9AA	0.7779	0.1132	0.1864	0.109*	
C10A	0.7467 (3)	0.1976 (2)	0.0741 (2)	0.0842 (8)	
H10A	0.8308	0.1903	0.0408	0.101*	
C11A	0.6548 (2)	0.25519 (17)	0.03618 (18)	0.0644 (5)	
H11A	0.6771	0.2870	−0.0222	0.077*	
C12A	0.44407 (17)	0.22803 (14)	−0.10297 (14)	0.0504 (4)	
C13A	0.4429 (2)	0.13644 (15)	−0.06638 (18)	0.0636 (5)	
H13A	0.4151	0.1241	−0.0019	0.076*	
C14A	0.4839 (3)	0.06240 (19)	−0.1273 (2)	0.0807 (8)	
H14A	0.4845	−0.0001	−0.1034	0.097*	
C15A	0.5233 (2)	0.0809 (2)	−0.2219 (2)	0.0815 (8)	
H15A	0.5499	0.0310	−0.2622	0.098*	
C16A	0.5238 (2)	0.1724 (2)	−0.25782 (19)	0.0791 (8)	
H16A	0.5509	0.1843	−0.3224	0.095*	
C17A	0.4838 (2)	0.24787 (17)	−0.19820 (16)	0.0631 (5)	
H17A	0.4840	0.3103	−0.2222	0.076*	
C18A	0.2489 (2)	0.45154 (15)	0.25945 (14)	0.0570 (5)	
C19A	0.1353 (3)	0.5041 (3)	0.2648 (2)	0.1044 (11)	
H19A	0.1243	0.5517	0.2191	0.125*	
C20A	0.0349 (4)	0.4859 (4)	0.3395 (3)	0.1405 (18)	
H20A	−0.0436	0.5216	0.3438	0.169*	
C21A	0.0515 (4)	0.4165 (3)	0.4055 (3)	0.1161 (13)	
H21A	−0.0166	0.4035	0.4543	0.139*	
C22A	0.1662 (4)	0.3658 (2)	0.4012 (2)	0.1059 (12)	
H22A	0.1775	0.3196	0.4481	0.127*	
C23A	0.2668 (3)	0.38203 (18)	0.32767 (18)	0.0828 (7)	
H23A	0.3454	0.3466	0.3243	0.099*	
S1B	0.10148 (4)	0.84479 (3)	0.50127 (3)	0.04992 (14)	
O1B	0.33919 (16)	0.96467 (12)	0.56269 (11)	0.0724 (4)	
O2B	0.00062 (15)	0.80155 (12)	0.46302 (11)	0.0701 (4)	
O3B	0.06872 (15)	0.89164 (12)	0.58981 (10)	0.0660 (4)	
N1B	0.17893 (14)	1.02892 (11)	0.29402 (10)	0.0456 (3)	
N2B	0.24738 (14)	1.06815 (11)	0.35475 (11)	0.0485 (3)	
C1B	0.24196 (16)	1.00752 (13)	0.42902 (12)	0.0455 (4)	
C2B	0.16822 (16)	0.92861 (13)	0.41561 (12)	0.0444 (4)	
C3B	0.12909 (16)	0.94527 (13)	0.32797 (12)	0.0432 (4)	
C4B	0.31394 (18)	1.02748 (15)	0.50764 (14)	0.0534 (4)	
C5B	0.3515 (3)	1.12733 (18)	0.51436 (19)	0.0755 (6)	
H5BA	0.4079	1.1297	0.5613	0.113*	
H5BB	0.2746	1.1674	0.5323	0.113*	
H5BC	0.3961	1.1491	0.4538	0.113*	
C6B	0.04661 (17)	0.88993 (13)	0.27640 (12)	0.0465 (4)	
C7B	0.0984 (2)	0.80847 (16)	0.23040 (16)	0.0618 (5)	
H7BA	0.1860	0.7901	0.2288	0.074*	
C8B	0.0189 (3)	0.75462 (18)	0.18691 (18)	0.0790 (7)	
H8BA	0.0531	0.7003	0.1552	0.095*	
C9B	−0.1115 (3)	0.7818 (2)	0.19072 (18)	0.0809 (7)	
H9BA	−0.1658	0.7440	0.1640	0.097*	
C10B	−0.1607 (2)	0.8637 (2)	0.23349 (18)	0.0761 (7)	
H10B	−0.2478	0.8827	0.2338	0.091*	
C11B	−0.08225 (19)	0.91856 (17)	0.27633 (15)	0.0591 (5)	
H11B	−0.1161	0.9746	0.3050	0.071*	
C12B	0.15416 (17)	1.08518 (15)	0.21182 (13)	0.0523 (4)	
C13B	0.1600 (2)	1.0446 (2)	0.12513 (15)	0.0695 (6)	
H13B	0.1821	0.9802	0.1172	0.083*	
C14B	0.1313 (3)	1.1044 (3)	0.04867 (17)	0.0887 (9)	
H14B	0.1322	1.0787	−0.0105	0.106*	
C15B	0.1021 (3)	1.1993 (2)	0.0597 (2)	0.0861 (8)	
H15B	0.0844	1.2378	0.0081	0.103*	
C16B	0.0988 (2)	1.2376 (2)	0.1462 (2)	0.0778 (7)	
H16B	0.0800	1.3025	0.1532	0.093*	
C17B	0.1231 (2)	1.18103 (16)	0.22353 (17)	0.0623 (5)	
H17B	0.1187	1.2072	0.2829	0.075*	
C18B	0.2234 (2)	0.75497 (14)	0.51379 (15)	0.0569 (5)	
C19B	0.2700 (3)	0.74521 (19)	0.59938 (19)	0.0809 (7)	
H19B	0.2442	0.7891	0.6465	0.097*	
C20B	0.3550 (4)	0.6699 (2)	0.6142 (3)	0.1172 (13)	
H20B	0.3855	0.6615	0.6720	0.141*	
C21B	0.3938 (5)	0.6080 (3)	0.5438 (3)	0.149 (2)	
H21B	0.4535	0.5584	0.5532	0.178*	
C22B	0.3466 (5)	0.6169 (3)	0.4582 (3)	0.1325 (16)	
H22B	0.3741	0.5736	0.4108	0.159*	
C23B	0.2583 (3)	0.69079 (18)	0.4439 (2)	0.0825 (8)	
H23B	0.2232	0.6968	0.3877	0.099*	

### Atomic displacement parameters (Å^2^)

**Table:**

	*U*^11^	*U*^22^	*U*^33^	*U*^12^	*U*^13^	*U*^23^
S1A	0.0575 (3)	0.0468 (3)	0.0485 (3)	−0.01179 (19)	−0.01181 (19)	−0.00416 (19)
O1A	0.0801 (11)	0.0753 (11)	0.0796 (11)	0.0198 (8)	−0.0109 (9)	−0.0205 (9)
O2A	0.0992 (12)	0.0461 (8)	0.0648 (9)	−0.0192 (7)	−0.0175 (8)	−0.0011 (6)
O3A	0.0643 (9)	0.0800 (10)	0.0671 (9)	−0.0079 (7)	−0.0251 (7)	−0.0113 (8)
N1A	0.0460 (8)	0.0446 (8)	0.0496 (8)	−0.0011 (6)	−0.0112 (6)	−0.0054 (6)
N2A	0.0465 (8)	0.0496 (9)	0.0531 (8)	−0.0022 (6)	−0.0140 (6)	−0.0038 (7)
C1A	0.0442 (9)	0.0476 (10)	0.0499 (10)	−0.0063 (7)	−0.0085 (7)	−0.0043 (8)
C2A	0.0459 (9)	0.0459 (9)	0.0444 (9)	−0.0073 (7)	−0.0072 (7)	−0.0035 (7)
C3A	0.0441 (9)	0.0456 (9)	0.0473 (9)	−0.0093 (7)	−0.0086 (7)	−0.0013 (7)
C4A	0.0503 (10)	0.0497 (11)	0.0678 (12)	−0.0027 (8)	−0.0148 (9)	−0.0038 (9)
C5A	0.0716 (14)	0.0804 (16)	0.0789 (15)	0.0166 (12)	−0.0268 (12)	0.0020 (12)
C6A	0.0528 (10)	0.0441 (10)	0.0569 (11)	−0.0063 (7)	−0.0180 (8)	−0.0019 (8)
C7A	0.0793 (14)	0.0526 (11)	0.0568 (11)	−0.0107 (10)	−0.0196 (10)	−0.0002 (9)
C8A	0.120 (2)	0.0578 (14)	0.0723 (15)	−0.0062 (13)	−0.0473 (15)	0.0048 (11)
C9A	0.102 (2)	0.0681 (16)	0.117 (2)	0.0136 (14)	−0.0672 (19)	−0.0055 (15)
C10A	0.0606 (13)	0.0802 (17)	0.117 (2)	0.0095 (12)	−0.0341 (14)	−0.0061 (16)
C11A	0.0523 (11)	0.0670 (13)	0.0765 (14)	−0.0035 (9)	−0.0184 (10)	0.0009 (11)
C12A	0.0408 (9)	0.0532 (11)	0.0594 (11)	−0.0023 (7)	−0.0111 (8)	−0.0154 (9)
C13A	0.0653 (12)	0.0557 (12)	0.0739 (13)	0.0052 (9)	−0.0236 (10)	−0.0133 (10)
C14A	0.0784 (15)	0.0606 (14)	0.110 (2)	0.0170 (11)	−0.0371 (15)	−0.0281 (14)
C15A	0.0543 (12)	0.0872 (19)	0.107 (2)	0.0106 (11)	−0.0152 (12)	−0.0520 (16)
C16A	0.0549 (12)	0.112 (2)	0.0703 (14)	−0.0170 (12)	0.0035 (10)	−0.0390 (14)
C17A	0.0577 (11)	0.0716 (14)	0.0615 (12)	−0.0158 (10)	−0.0061 (9)	−0.0159 (10)
C18A	0.0694 (12)	0.0529 (11)	0.0495 (10)	−0.0108 (9)	−0.0067 (9)	−0.0096 (8)
C19A	0.094 (2)	0.140 (3)	0.0670 (16)	0.0335 (19)	0.0079 (14)	0.0078 (17)
C20A	0.099 (3)	0.213 (5)	0.094 (3)	0.040 (3)	0.017 (2)	−0.004 (3)
C21A	0.119 (3)	0.136 (3)	0.084 (2)	−0.040 (2)	0.030 (2)	−0.020 (2)
C22A	0.154 (3)	0.0703 (17)	0.0792 (19)	−0.0168 (19)	0.028 (2)	0.0070 (14)
C23A	0.115 (2)	0.0582 (14)	0.0678 (15)	−0.0011 (13)	0.0067 (14)	0.0043 (11)
S1B	0.0474 (2)	0.0543 (3)	0.0478 (3)	−0.00010 (19)	−0.00929 (18)	0.00447 (19)
O1B	0.0791 (10)	0.0794 (11)	0.0653 (9)	0.0014 (8)	−0.0343 (8)	0.0017 (8)
O2B	0.0605 (8)	0.0804 (11)	0.0736 (10)	−0.0217 (7)	−0.0231 (7)	0.0213 (8)
O3B	0.0711 (9)	0.0729 (10)	0.0488 (8)	0.0107 (7)	0.0016 (6)	−0.0014 (7)
N1B	0.0442 (7)	0.0512 (9)	0.0424 (7)	−0.0013 (6)	−0.0101 (6)	−0.0003 (6)
N2B	0.0456 (8)	0.0523 (9)	0.0492 (8)	−0.0026 (6)	−0.0120 (6)	−0.0034 (7)
C1B	0.0415 (8)	0.0500 (10)	0.0456 (9)	0.0036 (7)	−0.0096 (7)	−0.0044 (7)
C2B	0.0408 (8)	0.0470 (10)	0.0453 (9)	0.0043 (7)	−0.0083 (7)	−0.0022 (7)
C3B	0.0391 (8)	0.0458 (9)	0.0445 (9)	0.0030 (7)	−0.0070 (7)	−0.0044 (7)
C4B	0.0474 (9)	0.0642 (12)	0.0507 (10)	0.0020 (8)	−0.0137 (8)	−0.0078 (9)
C5B	0.0828 (16)	0.0755 (15)	0.0771 (15)	−0.0128 (12)	−0.0342 (13)	−0.0123 (12)
C6B	0.0472 (9)	0.0517 (10)	0.0417 (9)	−0.0027 (7)	−0.0107 (7)	−0.0012 (7)
C7B	0.0668 (12)	0.0583 (12)	0.0632 (12)	0.0063 (9)	−0.0200 (10)	−0.0120 (10)
C8B	0.114 (2)	0.0614 (14)	0.0675 (14)	−0.0050 (13)	−0.0299 (14)	−0.0164 (11)
C9B	0.0913 (18)	0.0945 (19)	0.0668 (14)	−0.0345 (15)	−0.0312 (13)	−0.0069 (13)
C10B	0.0548 (12)	0.113 (2)	0.0660 (14)	−0.0139 (12)	−0.0227 (10)	−0.0072 (13)
C11B	0.0488 (10)	0.0756 (14)	0.0547 (11)	0.0011 (9)	−0.0139 (8)	−0.0075 (10)
C12B	0.0443 (9)	0.0660 (12)	0.0470 (10)	−0.0078 (8)	−0.0097 (7)	0.0088 (8)
C13B	0.0748 (14)	0.0862 (16)	0.0480 (11)	−0.0117 (12)	−0.0097 (10)	0.0034 (10)
C14B	0.0884 (17)	0.135 (3)	0.0470 (12)	−0.0330 (17)	−0.0173 (12)	0.0137 (14)
C15B	0.0750 (16)	0.106 (2)	0.0809 (18)	−0.0237 (15)	−0.0271 (13)	0.0418 (16)
C16B	0.0700 (14)	0.0780 (16)	0.0869 (18)	−0.0091 (12)	−0.0233 (12)	0.0289 (13)
C17B	0.0578 (11)	0.0610 (13)	0.0688 (13)	−0.0063 (9)	−0.0143 (10)	0.0105 (10)
C18B	0.0663 (12)	0.0448 (10)	0.0624 (12)	0.0025 (8)	−0.0211 (9)	−0.0005 (9)
C19B	0.1076 (19)	0.0654 (15)	0.0790 (16)	0.0189 (13)	−0.0490 (15)	−0.0101 (12)
C20B	0.156 (3)	0.088 (2)	0.125 (3)	0.042 (2)	−0.088 (3)	−0.0128 (19)
C21B	0.194 (4)	0.102 (3)	0.165 (4)	0.089 (3)	−0.099 (4)	−0.041 (3)
C22B	0.190 (4)	0.089 (2)	0.124 (3)	0.071 (3)	−0.058 (3)	−0.040 (2)
C23B	0.114 (2)	0.0610 (14)	0.0764 (15)	0.0210 (13)	−0.0317 (15)	−0.0121 (12)

### Geometric parameters (Å, º)

**Table:**

S1A—O2A	1.4239 (16)	S1B—O2B	1.4315 (16)
S1A—O3A	1.4286 (16)	S1B—O3B	1.4322 (16)
S1A—C2A	1.7550 (18)	S1B—C2B	1.7509 (18)
S1A—C18A	1.766 (2)	S1B—C18B	1.766 (2)
O1A—C4A	1.203 (3)	O1B—C4B	1.203 (3)
N1A—N2A	1.347 (2)	N1B—C3B	1.353 (2)
N1A—C3A	1.362 (2)	N1B—N2B	1.357 (2)
N1A—C12A	1.434 (2)	N1B—C12B	1.436 (2)
N2A—C1A	1.326 (2)	N2B—C1B	1.328 (2)
C1A—C2A	1.424 (3)	C1B—C2B	1.421 (3)
C1A—C4A	1.487 (3)	C1B—C4B	1.487 (2)
C2A—C3A	1.384 (3)	C2B—C3B	1.381 (2)
C3A—C6A	1.478 (3)	C3B—C6B	1.485 (2)
C4A—C5A	1.498 (3)	C4B—C5B	1.491 (3)
C5A—H5AA	0.9600	C5B—H5BA	0.9600
C5A—H5AB	0.9600	C5B—H5BB	0.9600
C5A—H5AC	0.9600	C5B—H5BC	0.9600
C6A—C11A	1.384 (3)	C6B—C11B	1.379 (3)
C6A—C7A	1.392 (3)	C6B—C7B	1.386 (3)
C7A—C8A	1.377 (3)	C7B—C8B	1.384 (3)
C7A—H7AA	0.9300	C7B—H7BA	0.9300
C8A—C9A	1.374 (4)	C8B—C9B	1.383 (4)
C8A—H8AA	0.9300	C8B—H8BA	0.9300
C9A—C10A	1.375 (5)	C9B—C10B	1.364 (4)
C9A—H9AA	0.9300	C9B—H9BA	0.9300
C10A—C11A	1.381 (3)	C10B—C11B	1.380 (3)
C10A—H10A	0.9300	C10B—H10B	0.9300
C11A—H11A	0.9300	C11B—H11B	0.9300
C12A—C13A	1.371 (3)	C12B—C13B	1.374 (3)
C12A—C17A	1.376 (3)	C12B—C17B	1.382 (3)
C13A—C14A	1.390 (3)	C13B—C14B	1.409 (4)
C13A—H13A	0.9300	C13B—H13B	0.9300
C14A—C15A	1.364 (4)	C14B—C15B	1.365 (5)
C14A—H14A	0.9300	C14B—H14B	0.9300
C15A—C16A	1.366 (4)	C15B—C16B	1.360 (4)
C15A—H15A	0.9300	C15B—H15B	0.9300
C16A—C17A	1.393 (3)	C16B—C17B	1.381 (3)
C16A—H16A	0.9300	C16B—H16B	0.9300
C17A—H17A	0.9300	C17B—H17B	0.9300
C18A—C19A	1.354 (4)	C18B—C23B	1.366 (3)
C18A—C23A	1.377 (3)	C18B—C19B	1.381 (3)
C19A—C20A	1.394 (5)	C19B—C20B	1.374 (4)
C19A—H19A	0.9300	C19B—H19B	0.9300
C20A—C21A	1.352 (6)	C20B—C21B	1.354 (5)
C20A—H20A	0.9300	C20B—H20B	0.9300
C21A—C22A	1.351 (6)	C21B—C22B	1.384 (5)
C21A—H21A	0.9300	C21B—H21B	0.9300
C22A—C23A	1.379 (4)	C22B—C23B	1.380 (4)
C22A—H22A	0.9300	C22B—H22B	0.9300
C23A—H23A	0.9300	C23B—H23B	0.9300
			
O2A—S1A—O3A	119.09 (10)	O2B—S1B—O3B	118.74 (10)
O2A—S1A—C2A	107.69 (9)	O2B—S1B—C2B	106.86 (9)
O3A—S1A—C2A	107.24 (9)	O3B—S1B—C2B	107.65 (9)
O2A—S1A—C18A	107.91 (10)	O2B—S1B—C18B	107.10 (10)
O3A—S1A—C18A	107.18 (10)	O3B—S1B—C18B	108.21 (10)
C2A—S1A—C18A	107.20 (9)	C2B—S1B—C18B	107.85 (9)
N2A—N1A—C3A	112.99 (14)	C3B—N1B—N2B	112.81 (14)
N2A—N1A—C12A	117.96 (15)	C3B—N1B—C12B	128.97 (15)
C3A—N1A—C12A	129.04 (15)	N2B—N1B—C12B	117.60 (15)
C1A—N2A—N1A	105.78 (14)	C1B—N2B—N1B	105.25 (15)
N2A—C1A—C2A	110.14 (16)	N2B—C1B—C2B	110.48 (15)
N2A—C1A—C4A	117.29 (16)	N2B—C1B—C4B	118.93 (17)
C2A—C1A—C4A	132.53 (18)	C2B—C1B—C4B	130.52 (17)
C3A—C2A—C1A	105.76 (16)	C3B—C2B—C1B	105.55 (16)
C3A—C2A—S1A	125.25 (14)	C3B—C2B—S1B	124.47 (14)
C1A—C2A—S1A	128.18 (14)	C1B—C2B—S1B	127.93 (14)
N1A—C3A—C2A	105.33 (15)	N1B—C3B—C2B	105.90 (15)
N1A—C3A—C6A	121.10 (16)	N1B—C3B—C6B	123.22 (15)
C2A—C3A—C6A	133.47 (17)	C2B—C3B—C6B	130.86 (17)
O1A—C4A—C1A	121.15 (19)	O1B—C4B—C1B	120.27 (19)
O1A—C4A—C5A	122.17 (19)	O1B—C4B—C5B	122.72 (19)
C1A—C4A—C5A	116.68 (18)	C1B—C4B—C5B	117.02 (18)
C4A—C5A—H5AA	109.5	C4B—C5B—H5BA	109.5
C4A—C5A—H5AB	109.5	C4B—C5B—H5BB	109.5
H5AA—C5A—H5AB	109.5	H5BA—C5B—H5BB	109.5
C4A—C5A—H5AC	109.5	C4B—C5B—H5BC	109.5
H5AA—C5A—H5AC	109.5	H5BA—C5B—H5BC	109.5
H5AB—C5A—H5AC	109.5	H5BB—C5B—H5BC	109.5
C11A—C6A—C7A	119.68 (19)	C11B—C6B—C7B	120.18 (19)
C11A—C6A—C3A	119.30 (18)	C11B—C6B—C3B	119.87 (17)
C7A—C6A—C3A	120.99 (18)	C7B—C6B—C3B	119.93 (16)
C8A—C7A—C6A	119.7 (2)	C8B—C7B—C6B	119.5 (2)
C8A—C7A—H7AA	120.1	C8B—C7B—H7BA	120.3
C6A—C7A—H7AA	120.1	C6B—C7B—H7BA	120.3
C9A—C8A—C7A	120.6 (2)	C9B—C8B—C7B	119.9 (2)
C9A—C8A—H8AA	119.7	C9B—C8B—H8BA	120.1
C7A—C8A—H8AA	119.7	C7B—C8B—H8BA	120.1
C8A—C9A—C10A	119.7 (2)	C10B—C9B—C8B	120.3 (2)
C8A—C9A—H9AA	120.1	C10B—C9B—H9BA	119.9
C10A—C9A—H9AA	120.1	C8B—C9B—H9BA	119.9
C9A—C10A—C11A	120.6 (3)	C9B—C10B—C11B	120.4 (2)
C9A—C10A—H10A	119.7	C9B—C10B—H10B	119.8
C11A—C10A—H10A	119.7	C11B—C10B—H10B	119.8
C10A—C11A—C6A	119.6 (2)	C6B—C11B—C10B	119.7 (2)
C10A—C11A—H11A	120.2	C6B—C11B—H11B	120.2
C6A—C11A—H11A	120.2	C10B—C11B—H11B	120.2
C13A—C12A—C17A	121.5 (2)	C13B—C12B—C17B	121.5 (2)
C13A—C12A—N1A	120.07 (19)	C13B—C12B—N1B	121.0 (2)
C17A—C12A—N1A	118.36 (19)	C17B—C12B—N1B	117.53 (18)
C12A—C13A—C14A	118.8 (2)	C12B—C13B—C14B	117.3 (3)
C12A—C13A—H13A	120.6	C12B—C13B—H13B	121.3
C14A—C13A—H13A	120.6	C14B—C13B—H13B	121.3
C15A—C14A—C13A	120.3 (3)	C15B—C14B—C13B	121.3 (3)
C15A—C14A—H14A	119.9	C15B—C14B—H14B	119.3
C13A—C14A—H14A	119.9	C13B—C14B—H14B	119.3
C14A—C15A—C16A	120.5 (2)	C16B—C15B—C14B	119.9 (2)
C14A—C15A—H15A	119.8	C16B—C15B—H15B	120.1
C16A—C15A—H15A	119.8	C14B—C15B—H15B	120.1
C15A—C16A—C17A	120.3 (3)	C15B—C16B—C17B	120.6 (3)
C15A—C16A—H16A	119.8	C15B—C16B—H16B	119.7
C17A—C16A—H16A	119.8	C17B—C16B—H16B	119.7
C12A—C17A—C16A	118.5 (2)	C16B—C17B—C12B	119.3 (2)
C12A—C17A—H17A	120.7	C16B—C17B—H17B	120.3
C16A—C17A—H17A	120.7	C12B—C17B—H17B	120.3
C19A—C18A—C23A	120.7 (2)	C23B—C18B—C19B	121.6 (2)
C19A—C18A—S1A	120.0 (2)	C23B—C18B—S1B	119.67 (17)
C23A—C18A—S1A	119.15 (19)	C19B—C18B—S1B	118.33 (18)
C18A—C19A—C20A	119.2 (3)	C20B—C19B—C18B	119.3 (3)
C18A—C19A—H19A	120.4	C20B—C19B—H19B	120.3
C20A—C19A—H19A	120.4	C18B—C19B—H19B	120.3
C21A—C20A—C19A	120.1 (4)	C21B—C20B—C19B	119.4 (3)
C21A—C20A—H20A	120.0	C21B—C20B—H20B	120.3
C19A—C20A—H20A	120.0	C19B—C20B—H20B	120.3
C22A—C21A—C20A	120.4 (3)	C20B—C21B—C22B	121.6 (3)
C22A—C21A—H21A	119.8	C20B—C21B—H21B	119.2
C20A—C21A—H21A	119.8	C22B—C21B—H21B	119.2
C21A—C22A—C23A	120.6 (3)	C23B—C22B—C21B	119.3 (3)
C21A—C22A—H22A	119.7	C23B—C22B—H22B	120.3
C23A—C22A—H22A	119.7	C21B—C22B—H22B	120.3
C18A—C23A—C22A	118.9 (3)	C18B—C23B—C22B	118.7 (3)
C18A—C23A—H23A	120.6	C18B—C23B—H23B	120.6
C22A—C23A—H23A	120.6	C22B—C23B—H23B	120.6
			
C3A—N1A—N2A—C1A	−0.7 (2)	C3B—N1B—N2B—C1B	0.52 (19)
C12A—N1A—N2A—C1A	−179.55 (16)	C12B—N1B—N2B—C1B	172.29 (15)
N1A—N2A—C1A—C2A	0.3 (2)	N1B—N2B—C1B—C2B	−0.33 (19)
N1A—N2A—C1A—C4A	−177.81 (16)	N1B—N2B—C1B—C4B	177.02 (15)
N2A—C1A—C2A—C3A	0.2 (2)	N2B—C1B—C2B—C3B	0.05 (19)
C4A—C1A—C2A—C3A	177.90 (19)	C4B—C1B—C2B—C3B	−176.91 (17)
N2A—C1A—C2A—S1A	170.16 (14)	N2B—C1B—C2B—S1B	−164.01 (13)
C4A—C1A—C2A—S1A	−12.1 (3)	C4B—C1B—C2B—S1B	19.0 (3)
O2A—S1A—C2A—C3A	140.84 (17)	O2B—S1B—C2B—C3B	−1.34 (18)
O3A—S1A—C2A—C3A	11.55 (19)	O3B—S1B—C2B—C3B	−129.93 (15)
C18A—S1A—C2A—C3A	−103.27 (17)	C18B—S1B—C2B—C3B	113.51 (16)
O2A—S1A—C2A—C1A	−27.3 (2)	O2B—S1B—C2B—C1B	159.94 (16)
O3A—S1A—C2A—C1A	−156.63 (17)	O3B—S1B—C2B—C1B	31.35 (18)
C18A—S1A—C2A—C1A	88.55 (18)	C18B—S1B—C2B—C1B	−85.21 (17)
N2A—N1A—C3A—C2A	0.8 (2)	N2B—N1B—C3B—C2B	−0.50 (19)
C12A—N1A—C3A—C2A	179.49 (17)	C12B—N1B—C3B—C2B	−171.11 (17)
N2A—N1A—C3A—C6A	−176.07 (16)	N2B—N1B—C3B—C6B	177.85 (15)
C12A—N1A—C3A—C6A	2.6 (3)	C12B—N1B—C3B—C6B	7.2 (3)
C1A—C2A—C3A—N1A	−0.56 (19)	C1B—C2B—C3B—N1B	0.26 (18)
S1A—C2A—C3A—N1A	−170.93 (13)	S1B—C2B—C3B—N1B	165.03 (12)
C1A—C2A—C3A—C6A	175.76 (19)	C1B—C2B—C3B—C6B	−177.91 (17)
S1A—C2A—C3A—C6A	5.4 (3)	S1B—C2B—C3B—C6B	−13.1 (3)
N2A—C1A—C4A—O1A	155.7 (2)	N2B—C1B—C4B—O1B	−160.87 (19)
C2A—C1A—C4A—O1A	−21.9 (3)	C2B—C1B—C4B—O1B	15.9 (3)
N2A—C1A—C4A—C5A	−23.8 (3)	N2B—C1B—C4B—C5B	19.4 (3)
C2A—C1A—C4A—C5A	158.6 (2)	C2B—C1B—C4B—C5B	−163.9 (2)
N1A—C3A—C6A—C11A	59.4 (2)	N1B—C3B—C6B—C11B	−76.8 (2)
C2A—C3A—C6A—C11A	−116.5 (2)	C2B—C3B—C6B—C11B	101.1 (2)
N1A—C3A—C6A—C7A	−118.7 (2)	N1B—C3B—C6B—C7B	104.7 (2)
C2A—C3A—C6A—C7A	65.5 (3)	C2B—C3B—C6B—C7B	−77.4 (3)
C11A—C6A—C7A—C8A	1.3 (3)	C11B—C6B—C7B—C8B	−1.9 (3)
C3A—C6A—C7A—C8A	179.28 (19)	C3B—C6B—C7B—C8B	176.5 (2)
C6A—C7A—C8A—C9A	−0.8 (4)	C6B—C7B—C8B—C9B	−0.8 (4)
C7A—C8A—C9A—C10A	−0.4 (4)	C7B—C8B—C9B—C10B	2.9 (4)
C8A—C9A—C10A—C11A	1.1 (4)	C8B—C9B—C10B—C11B	−2.3 (4)
C9A—C10A—C11A—C6A	−0.6 (4)	C7B—C6B—C11B—C10B	2.6 (3)
C7A—C6A—C11A—C10A	−0.6 (3)	C3B—C6B—C11B—C10B	−175.9 (2)
C3A—C6A—C11A—C10A	−178.6 (2)	C9B—C10B—C11B—C6B	−0.5 (4)
N2A—N1A—C12A—C13A	−131.36 (19)	C3B—N1B—C12B—C13B	−49.0 (3)
C3A—N1A—C12A—C13A	50.0 (3)	N2B—N1B—C12B—C13B	140.82 (19)
N2A—N1A—C12A—C17A	46.2 (2)	C3B—N1B—C12B—C17B	130.4 (2)
C3A—N1A—C12A—C17A	−132.4 (2)	N2B—N1B—C12B—C17B	−39.8 (2)
C17A—C12A—C13A—C14A	0.5 (3)	C17B—C12B—C13B—C14B	−0.9 (3)
N1A—C12A—C13A—C14A	178.02 (19)	N1B—C12B—C13B—C14B	178.5 (2)
C12A—C13A—C14A—C15A	−0.7 (3)	C12B—C13B—C14B—C15B	1.7 (4)
C13A—C14A—C15A—C16A	0.5 (4)	C13B—C14B—C15B—C16B	−0.8 (4)
C14A—C15A—C16A—C17A	−0.1 (4)	C14B—C15B—C16B—C17B	−0.9 (4)
C13A—C12A—C17A—C16A	−0.2 (3)	C15B—C16B—C17B—C12B	1.6 (4)
N1A—C12A—C17A—C16A	−177.69 (18)	C13B—C12B—C17B—C16B	−0.7 (3)
C15A—C16A—C17A—C12A	0.0 (3)	N1B—C12B—C17B—C16B	179.91 (19)
O2A—S1A—C18A—C19A	27.2 (3)	O2B—S1B—C18B—C23B	41.4 (2)
O3A—S1A—C18A—C19A	156.6 (2)	O3B—S1B—C18B—C23B	170.6 (2)
C2A—S1A—C18A—C19A	−88.6 (3)	C2B—S1B—C18B—C23B	−73.3 (2)
O2A—S1A—C18A—C23A	−149.70 (19)	O2B—S1B—C18B—C19B	−131.2 (2)
O3A—S1A—C18A—C23A	−20.3 (2)	O3B—S1B—C18B—C19B	−2.1 (2)
C2A—S1A—C18A—C23A	94.6 (2)	C2B—S1B—C18B—C19B	114.1 (2)
C23A—C18A—C19A—C20A	−1.2 (5)	C23B—C18B—C19B—C20B	0.9 (5)
S1A—C18A—C19A—C20A	−178.0 (3)	S1B—C18B—C19B—C20B	173.4 (3)
C18A—C19A—C20A—C21A	−0.1 (7)	C18B—C19B—C20B—C21B	1.6 (6)
C19A—C20A—C21A—C22A	1.7 (7)	C19B—C20B—C21B—C22B	−2.2 (8)
C20A—C21A—C22A—C23A	−2.1 (6)	C20B—C21B—C22B—C23B	0.3 (9)
C19A—C18A—C23A—C22A	0.8 (4)	C19B—C18B—C23B—C22B	−2.8 (5)
S1A—C18A—C23A—C22A	177.6 (2)	S1B—C18B—C23B—C22B	−175.2 (3)
C21A—C22A—C23A—C18A	0.8 (5)	C21B—C22B—C23B—C18B	2.2 (7)

### Hydrogen-bond geometry (Å, º)

**Table:**

*D*—H···*A*	*D*—H	H···*A*	*D*···*A*	*D*—H···*A*
C11*A*—H11*A*···O2*A*^i^	0.93	2.59	3.428 (3)	149
C11*B*—H11*B*···O3*B*^ii^	0.93	2.57	3.370 (3)	144

Symmetry codes: (i) −*x*+1, −*y*+1, −*z*; (ii) −*x*, −*y*+2, −*z*+1.

## References

[bb1] Bruker (2009). *APEX2*, *SAINT* and *SADABS* Bruker AXS Inc., Madison, Wisconsin, USA.

[bb2] Gürsoy, A., Demirayak, S., Capan, G., Erol, K. & Vural, K. (2000). *Eur. J. Med. Chem.* **35**, 359–364. 10.1016/s0223-5234(00)00117-3 10785562

[bb3] Kurumbail, R. G., Stevens, A. M., Gierse, J. K., McDonald, J. J., Stegeman, R. A., Pak, J. Y., Gildehaus, D. M., iyashiro, J., Penning, T. D., Seibert, K. C., Isakson, P. & Stallings, W. C. (1996). *Nature (London)*, **384**, 644–648. 10.1038/384644a0 8967954

[bb4] Nassar, E., Abdel-Aziz, H. A., Ibrahim, H. S. & Mansour, A. M. (2011). *Sci. Pharm.* **79**, 507–524. 10.3797/scipharm.1105-14 PMC3163377 21886900

[bb5] Saleh, T. S. & Abd El-Rahman, N. M. (2009). *Ultrason. Sonochem.* **16**, 237–242. 10.1016/j.ultsonch.2008.07.012 18835210

[bb6] Sheldrick, G. M. (2008). *Acta Cryst.* A**64**, 112–122. 10.1107/S0108767307043930 18156677

[bb7] Spek, A. L. (2009). *Acta Cryst.* D**65**, 148–155. 10.1107/S090744490804362X PMC2631630 19171970

